# Transcriptional responses underlying the hormetic and detrimental effects of the plant secondary metabolite gossypol on the generalist herbivore *Helicoverpa armigera*

**DOI:** 10.1186/1471-2164-12-575

**Published:** 2011-11-23

**Authors:** Maria de la Paz Celorio-Mancera, Seung-Joon Ahn, Heiko Vogel, David G Heckel

**Affiliations:** 1 Max Planck Institute for Chemical Ecology, Department of Entomology, Beutenberg Campus, Hans-Knöll-Straβe 8, 07745, Jena, Germany; 2 Department of Zoology Ecology, Stockholm University, Svante Arrheniusväg 18 B, 106 91, Stockholm, Sweden; 3 Horticultural and Herbal Crop Environment Division, National Institute of Horticultural and Herbal Science, Rural Development Administration, Suwon, 441-440, Korea

## Abstract

**Background:**

Hormesis is a biphasic biological response characterized by the stimulatory effect at relatively low amounts of chemical compounds which are otherwise detrimental at higher concentrations. A hormetic response in larval growth rates has been observed in cotton-feeding insects in response to increasing concentrations of gossypol, a toxic metabolite found in the pigment glands of some plants in the family Malvaceae. We investigated the developmental effect of gossypol in the cotton bollworm, *Helicoverpa armigera*, an important heliothine pest species, by exposing larvae to different doses of this metabolite in their diet. In addition, we sought to determine the underlying transcriptional responses to different gossypol doses.

**Results:**

Larval weight gain, pupal weight and larval development time were measured in feeding experiments and a hormetic response was seen for the first two characters. On the basis of net larval weight gain responses to gossypol, three concentrations (0%, 0.016% and 0.16%) were selected for transcript profiling in the gut and the rest of the body in a two-color double reference design microarray experiment. Hormesis could be observed at the transcript level, since at the low gossypol dose, genes involved in energy acquisition such as β-fructofuranosidases were up-regulated in the gut, and genes involved in cell adhesion were down-regulated in the body. Genes with products predicted to be integral to the membrane or associated with the proteasome core complex were significantly affected by the detrimental dose treatment in the body. Oxidoreductase activity-related genes were observed to be significantly altered in both tissues at the highest gossypol dose.

**Conclusions:**

This study represents the first transcriptional profiling approach investigating the effects of different concentrations of gossypol in a lepidopteran species. *H. armigera*'s transcriptional response to gossypol feeding is tissue- and dose-dependent and involves diverse detoxifying mechanisms not only to alleviate direct effects of gossypol but also indirect damage such as pH disturbance and oxygen radical formation. Genes discovered through this transcriptional approach may be additional candidates for understanding gossypol detoxification and coping with gossypol-induced stress. In a generalist herbivore that has evolved transcriptionally-regulated responses to a variety of different plant compounds, hormesis may be due to a lower induction threshold of growth-promoting, stress-coping responses and a higher induction threshold of detoxification pathways that are costly and cause collateral damage to the cell.

## Background

Gossypol is a polyphenolic binaphthyl dialdehyde found in the pigment glands of plant species belonging to Malvaceae, most notably cotton, *Gossypium *spp. [1]. The toxicity of gossypol in cottonseed meal has spurred a multitude of studies of its effects on diverse biological systems. The presence of both phenolic and carbonyl groups in the gossypol structure allows the interaction with other molecules through a wide range of weak to strong chemical bonds. This compound can covalently bind to amino acids, particularly lysine, through Schiff's base condensation reactions, and its dimeric structure facilitates cross-linking of proteins. Gossypol can also chelate iron and other metal ions and be both a pro-oxidant and antioxidant [2,3]. Moreover, the aromatic rings in gossypol render it a fairly hydrophobic compound able to penetrate biological membranes more readily [4]. All this intricate chemical reactivity implies the existence of multiple targets, which is reflected by the different enzyme classes inhibited by gossypol, especially those involved in energy production processes of the cell, and in the variety of biological effects attributed to this compound due to its antitumor, spermicidal, antimalarial, antiparasitic, antiamoebic and antiviral activities [3,5].

There is only limited knowledge regarding the mode of action of gossypol regardless of its therapeutic or toxic effect on different biological systems. It is clear, however, that the mechanism mostly relies on the disturbance of proteins and membranes. In addition, gossypol occurs in two optically active forms, of which the (-)-enantiomer has been found to be more reactive towards biological systems, particularly non-ruminant animals. In fact, the molecular mechanism behind (-)-gossypol antineoplastic activity appears to rely on its interaction with antiapoptotic proteins in the outer mitochondrial membrane [6]. However, the inhibitory effect of gossypol on insects and fungi is not enantiospecific [7]. Therefore, the ability to extrapolate across systems is limited and the response within a given system is likely to be very complex and dose-dependent.

Both quantity and quality of gossypol in plant tissues vary according to several factors such as cultivar, phenology or plant organ [1,8-10]. The protective nature of this sesquiterpene dimer to cotton against different insect herbivores became evident after the development of glandless cotton lines [3]. These lines were produced by conventional breeding to reduce the amount of gossypol in cottonseed meal which is fed to livestock. Some members of the "major-pest lineage" within the subfamily Heliothinae (Lepidoptera: Noctuidae) [11], prefer glandless cotton varieties over glanded ones [12]. Moreover, high concentrations of gossypol inhibit growth and development of tobacco budworm, *Heliothis virescens *[13]. However, the dose response is not monotonic and in fact gossypol acts as a hormetic agent, since the maximum weight gain was observed when larvae fed on a low dose gossypol-containing diet (0.0125%). Hormesis, first described in the late 1800's, is a widespread biological response towards an environmental stressor which is stimulatory in low amounts (e.g. increased fertility), and detrimental in high amounts (e.g. toxicity) [14].

Although the implications of the hormetic effect in the area of human toxicology remains controversial [15], there is substantial research regarding hormesis due to environmental stressors and their impact on aging and longevity in invertebrate species [16], and the efforts to understand the biology underlying the phenomenon continue [17]. Recent analyses for two animal systems (mammal and annelid) inquired whether gene expression in a global scale differs under the "low-dose effect" of toxic chemicals [18,19]. Dose-dependent transcriptional responses to gossypol have been observed for a handful of genes including the expression of mitochondrial-related genes in rat liver cells [20] and P450 monooxygenases in insects [21,22]. However, there is no unbiased transcriptional profiling approach documented investigating the effects of different doses of gossypol.

The cotton bollworm (CBW), *H. armigera*, is a generalist herbivore and one of the most injurious insect pests in the world, damaging a large number of plant species [23] with cotton as one of its most suitable hosts [24]. We investigated whether a hormetic effect of gossypol can be observed in *H. armigera *by measuring developmental parameters in response to different concentrations in the diet. In addition, we investigated the transcriptional response of *H. armigera *gut as well as the rest of body towards different dosages of gossypol using microarray expression profile analysis. The results herein provide a general view of the underlying transcriptional response to gossypol with implications for the detoxification of this plant secondary metabolite. They also indicate the role hormesis may play in the adaptation of generalist herbivores to a variety of hostplants presenting different sets of chemical challenges.

## Methods

### Insect rearing

*H. armigera *larvae were collected from Toowoomba, Queensland, Australia, in 2003 and reared on artificial diet under laboratory conditions (26°C, 55% RH, 16:8 hr = L:D) in Jena, Germany. The colony was maintained for 25 generations prior to the start of this study, exclusively by about 50 single-pair crosses per generation, avoiding brother-sister mating to minimize inbreeding. The artificial diet for larval rearing was purchased from BioServ (F9772, Frenchtown, NJ, USA). Gossypol from cotton seeds (G8761, racemic mixture, Sigma-Aldrich, MO, USA) was incorporated into the artificial diet using a carrier material of non-nutritive cellulose, "Alphacel", as reported elsewhere [25] in various concentrations (CT = 0.0, T1 = 0.0004, T2 = 0.0016, T3 = 0.004, T4 = 0.008, T5 = 0.016, T6 = 0.04, or T7 = 0.16% (w/v)). Cotton seeds contain a racemic mixture of gossypol with generally a higher percentage of the (+)-enantiomer although this may differ depending on the cotton species and cultivar [9].

### Gossypol treatment and larval development

A total of nine hundred and sixty larvae from a pool of newly molted fifth instar larvae were evenly divided into eight groups according to their initial fresh weights (I). Each group of larvae was randomly assigned to each gossypol treatment. Control group of larvae were exposed to the diet supplemented only with the Alphacel carrier (CT). Each group of larvae was composed of four biological replicates of thirty larvae. After those larvae were subjected to each treatment for three days, larvae were individually weighed as a measure of their final fresh weight (F), in order to calculate the net weight gain (= F-I).

Mortality was observed in CT, T4 and T7 treatments (4, 3 and 5 larvae were dead, respectively). Only one individual died in each T1 and T6, while no mortality was observed in the remaining treatments. Ten randomly chosen larvae from each replicate per treatment were separated for microarray analysis (see below), the other eighty larvae (20 larvae × 4 replicates) were returned to the same diet treatments and further observed for other developmental parameters: larval development time to pupation (day) and pupal weight in a day after pupation (mg). The developmental parameter data were analyzed by ANOVA and the statistical differences among treatment means were further tested by Post-hoc analysis.

### Expressed Sequence Tag (EST) Project

RNA was extracted from several larval tissues (e.g. midgut, fat body, integument), all larval instars and developmental stages (larvae and adults) with TRIzol Reagent (Invitrogen) according to the manufacturer's protocol. An additional DNAse (Turbo DNAse, Ambion) treatment was included to eliminate any contaminating DNA. The DNAse enzyme was removed and the RNA was further purified by using the RNeasy MinElute Cleanup Kit (Qiagen) following the manufacturer's protocol. RNA integrity and quantity was verified on an Agilent 2100 Bioanalyzer using the RNA Nano chips (Agilent Technologies). RNA quantity was determined on a Nanodrop ND-1000 spectrophotometer. Normalized full length-enriched cDNA libraries were generated using a combination of the SMART cDNA library construction kit and the Trimmer Direct cDNA normalization kit (Evrogen) following the manufacturer's protocol with several modifications [26]. The normalization process facilitates the identification of low abundance transcripts.

Single-pass sequencing of the 5' termini of cDNA library plasmid clones was carried out on an ABI 3730 xl automatic DNA sequencer (PE Applied Biosystems). Vector clipping, quality trimming and sequence assembly was done with the Lasergene software package (DNAStar Inc.). In total, 8 different cDNA libraries were generated from the tissues and developmental stages as described above and ~60,000 clones were sequenced. Additional sequencing was performed with a mixed cDNA pool on a Roche 454 FLX instrument, obtaining 274,607 high quality reads after trimming and quality filtering steps. The *H. armigera *ESTs generated and all publicly available Genbank sequences for this species were jointly assembled using Seqman NGen (Lasergene) and clustered into 27,381 contigs (putative gene objects) subsequently used for microarray oligo probe design.

### Microarray Design, labeling, hybridization and data acquisition

In order to optimize our *H. armigera *microarray design and maximize the output of subsequent gene expression profiling experiments, a Pre Selection Strategy (PSS, Imagenes) approach was used to select well performing probes based on initial test hybridizations. For the preliminary large array design each gene was tiled by a maximum number of probes. A total of 231,399 oligos for the 27,381 contigs were designed and a 244 K Agilent microarray was hybridized with labeled complex total RNA mixture and genomic DNA. The best performing probes for each gene were selected, for the expressed genes based on the RNA hybridization and for the non-expressed genes based on the DNA hybridization. A final condensed Agilent 4 × 44 K array design based on the eArray platform (Agilent Technologies; https://earray.chem.agilent.com/earray/) contains the few best performing probes of each gene (1-2 for each Gene Object) with a final number of 43803 non-control probe set and 1417 Agilent Technologies built in controls (structural and spike in).

A subset of forty ice-cold anesthetized larvae per treatment (having fed on 0%, 0.016%, or 0.16% gossypol diet for three days) was dissected longitudinally under phosphate-buffered saline and gut tissues (G) were separated from the rest of the body (RB). Four biological replicates were prepared by pooling ten individuals in each replicate of either G or RB tissues, snap-frozen in liquid nitrogen, and stored at -80°C until RNA isolation. Total RNA was purified, quality tested and quantified as mentioned above. Agilent Technologies spike-in RNA was added to 500 ng of total RNA and labelled using the Low RNA Input Linear Amplification kit (Agilent Technologies). Treated RNA and control samples were labelled with cyanine 5-CTP and 3-CTP dyes according to manufacturer instructions following a double reference dye-swap design. Labelled amplified cRNA samples were purified using RNeasy MinElute Cleanup kit (Qiagen) and analyzed on a Nanodrop spectrophotometer using the microarray function. Amplified cRNA samples were used for microarray hybridization only if the yield is > 825 ng and the specific activity is > 8.0 pmol Cy3 or Cy5 per ug cRNA. 825 ng each of cyanine 3 and cyanine5 labeled cRNA were used for each array. Hybridization was carried out at 65°C for 17 hours. Slides were washed in GE Wash Buffer 1 (Agilent Technologies) for 1 min at room temperature and a further minute in GE Wash Buffer 2 pre-warmed overnight to 37°C. Slides were treated in stabilization and drying solution (Agilent Technologies), scanned with the Agilent Microarray Scanner, and data was extracted from the TIFF images with Agilent Feature Extraction software version 9.1. The initial technical validation included visual inspection of images to identify gross abnormalities or background. Prior to normalization the sensitivity of the array and relationship between RNA concentration and fluorescent signal was assessed by calculating the signal intensity generated by reporters complementary to 10 'alien' synthetic RNA spikes introduced at known concentrations (from 1 pmole to 30 nmole prior to labeling). The microarray data reported in this paper have been deposited in the Gene Expression Omnibus (GEO) database, http://www.ncbi.nlm.nih.gov/geo [GEO:GPL14736].

### Microarray analysis

Expression profiling of *H. armigera *G and RB samples subjected to different gossypol-containing diets was generated by normalizing fluorescence signals to the median intensity and log base 2-transformation of the normalized data. In order to determine the relationship between the samples per tissue, the clustering application (Euclidean distance, average linkage) was applied to normalized to median, log-transformed, statistically significant data after ANOVA (unequal variance, no threshold, Benjamini and Hochberg false discovery rate (B&H FDR) multiple test correction, adjusted P cut off < 0.001) using the Geospiza GeneSifter^® ^genetic analysis software. Data was also filtered by volcano plots comparing each gossypol dosage to its control per tissue treatment by means of an unpaired t-test, unequal variance using Agilent GeneSpring GX11.5.1 software. All 43863 probes passed the data quality filtering based on intensity measurements. Only probes with corrected P values less than 0.001 after B&H FDR were considered statistically differentially expressed. Gene Ontology (GO) [27] annotations (for the target genes represented in the microarray) obtained through Blast2go (E-value cutoff: 0.001) [28] software were used to find significant GO terms (P < 0.001) represented in the statistically filtered data using GeneSpring. In order to perform a gene level analysis using GeneSpring program, the probes of the microarray along with the target EST sequences were assembled using Sequencher 4.7, contigs were blasted to public and in-house EST databases to assign correct gene codes to each probe. Nomenclature for important *H. armigera *detoxification genes such as esterases, cytochrome P450 s (P450 s), UDP-glycosyltransferases (UGTs) and glutathione transferases (GSTs) was included using public databases or by assigning it based on homology. Nomenclature for P450 s and UGTs was approved by the relevant Nomenclature Committees. Based on a list of homologous genes, we also inspected *Drosophila melanogaster *pathway enrichment in the data set for each t-test comparison using GeneSifter^® ^applying z-score statistics to determine whether a pathway occurs more or less frequently than expected as previously described by others [29]. The pathway source originated from the Kyoto Encyclopaedia of Genes and Genomes (KEGG) (Kanehisa Laboratories). Homology was established by obtaining best BLAST [30] hits (about 24% of the probes had a hit at e < 5 × 10-2) for *H. armigera *ESTs to *D. melanogaster *reviewed reference sequences (RefSeq) (See additional file 1: Table S1 displaying best RefSeq homolog hits to *H. armigera*). Homology databases containing the protein and nucleotide refseq accession were prepared from NCBI Entrez. In addition, the normalized log-ratios for each gene in all eight biological replicates per treatment comparison (relative to control), were used to apply the rank products (RP) method which has been proven to be a powerful method for identifying biologically relevant gene expression changes [31].

### Quantitative real-time PCR

Single stranded DNA from 500 ng of total RNA was obtained and amplified using Verso SYBR^® ^Green 2-Step QRT-PCR kit (Thermo Fisher Scientific) following manufacturer's instructions. Real-time PCR oligonucleotide primers were designed on the basis of sequences obtained for *H. armigera *CYP6AE14 and CYP6AE11 and two additional genes used as normalizers (endogenous control genes) i. e. 18 S ribosomal RNA and elongation initiation factor 4. Data was analyzed using the qBase 1.3.1 software (Biogazelle) and graphed as fold expression relative to the lowest expression across treatments.

## Results

### Effect of gossypol on larval development

**Figure 1:**
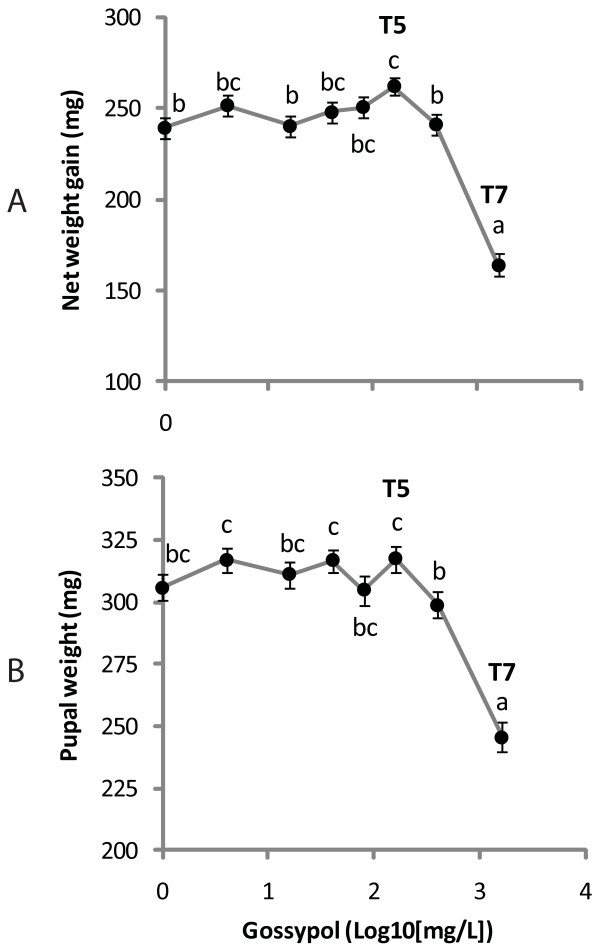
**Hormetic effect of gossypol on larval development**. Net larval weight gain (mg) (A) and pupal weight recorded one day after pupation (mg) (B) are plotted against the logarithm of gossypol concentration (mg/L). Means that are not indicated with the same letter are significantly different from each other as determined by post-hoc Duncan tests (P < 0.05) (feeding treatments T5 = 0.016%; T7 = 0.16% gossypol in insect diet).

When fifth-instar larvae were fed an artificial diet with different concentrations of gossypol, the highest net weight gain was observed at 0.016% (T5, Duncan's P < 0.05, Figure 1-A), a gain 10% greater than when gossypol was absent. Weight gain then steeply declined as gossypol concentration further increased, with the lowest gain occurring at 0.16% gossypol (T7, Figure 1-A). Pupal weight was also highest at T5, although not significantly higher than weights at lower doses (Figure 1-B). The lowest value for pupal weight and the longest time to pupation (See additional file 2: Figure S1 showing the effect of gossypol on larval developmental time to pupation) occurred at T7 with no significant differences between the remaining treatments for these two parameters.

### Effect of gossypol on gene expression

Since larval net weight gain was affected significantly only at the low T5 and high T7 gossypol doses, the transcriptional profiles of gut tissue (G) and the rest of the body (RB) from larvae collected from these treatments were examined by microarray analysis in a two-color double reference design using the 0% gossypol tissue samples as reference (See additional file 3: Figure S2-A depicting the microarray two-color design). Sample clustering using the expression profiles of the probes which passed the statistical filtering in GeneSifter yielded dendrograms grouping the control (CT) and T5 treatments closer to each other within each tissue type (See additional file 3: Figure S2-B depicting the hierarchical clustering of transcriptional responses across gossypol dose-tissue conditions).

#### Gene Ontology and KEGG Pathways Analyses

**Figure 2:**
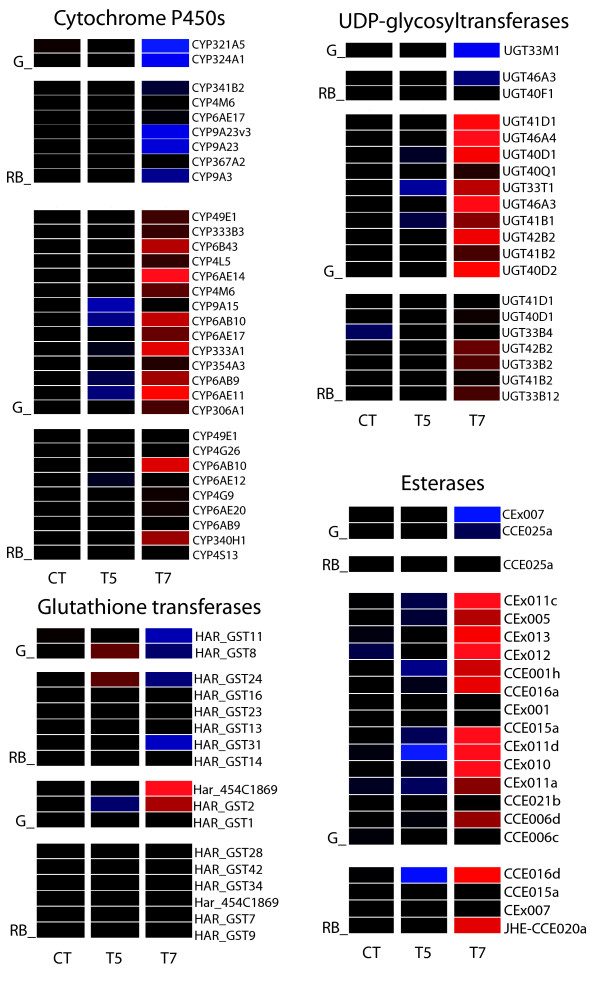
**Expression of detoxification gene families across gossypol dose-tissue experimental conditions**. Transcriptional responses of cytochrome P450 s, esterases, UDP-glycosyltransferases and glutathione transferases in gut and the rest of the body are displayed for two gossypol concentrations (CT: 0%; T5: 0.016%; T7: 0.16%). Responses are expressed relative to the median intensity of all data (blue = down-regulation; black = no change; red = up-regulation). Genes were found to be differentially expressed in *H. armigera *rest of body (RB) larval tissue to a 0.16% (T7) gossypol in the diet relative to the control samples (Welch's t-test, B&H FDR P < 0.001).

Genes found to be differentially down and up-regulated using t-test statistics were grouped according to Gene Ontology (GO) categories for comparisons and those GO categories significantly enriched across treatments are listed in Table 1. No GO category was significantly enriched in G-T5 (gut at 0.016% gossypol) with only one up-regulated EST for this condition, putatively encoding a glucan hydrolase based on the database Blast2go search. However this low gossypol concentration had a significant effect on the expression of genes involved in cell adhesion in the RB. The ESTs under this category were down-regulated and are homologous to members of the cadherin superfamily (See additional file 4: Table S2) while two non-annotated ESTs and one predicted to encode a product involved in fatty acid biosynthesis were up-regulated. Gene families potentially involved in detoxification of xenobiotics (cytochromes P450, UDP-glycosyltransferases, carboxylesterases, and glutathione transferases) were either unaffected or slightly down-regulated at T5, with the exception of one GST in the gut and one in the rest of the body (Figure 2).

**Table 1:** GO categories enriched in microarray comparisons through gossypol-tissue treatments

	GO accession	GO term	Corrected p-value
**T7- Gut/UP**			
	GO:0055114	oxidation reduction	7.19E-19
	GO:0016491	oxidoreductase activity	1.94E-18
	GO:0044429	mitochondrial part	4.01E-15
	GO:0005739	mitochondrion	2.28E-14
	GO:0005740	mitochondrial envelope	1.50E-10
	GO:0031966	mitochondrial membrane	2.77E-09
	GO:0003824	catalytic activity	6.22E-08
	GO:0031967	organelle envelope	6.77E-08
	GO:0005743	mitochondrial inner membrane	7.14E-08
	GO:0019866	organelle inner membrane	7.76E-08
	GO:0031975	envelope	5.36E-07
	GO:0031090	organelle membrane	7.13E-07
	GO:0044444	cytoplasmic part	6.98E-05
	GO:0015992	proton transport	7.52E-05
	GO:0006818	hydrogen transport	8.16E-05
	GO:0006091	generation of precursor metabolites and energy	8.16E-05
	GO:0042180	cellular ketone metabolic process	1.27E-04
	GO:0015077	monovalent inorganic cation transmembrane transporter activity	3.16E-04
	GO:0019752	carboxylic acid metabolic process	3.50E-04
	GO:0043436	oxoacid metabolic process	3.50E-04
	GO:0006082	organic acid metabolic process	3.50E-04
	GO:0005759	mitochondrial matrix	4.27E-04
	GO:0031980	mitochondrial lumen	4.27E-04
	GO:0008610	lipid biosynthetic process	4.81E-04
	GO:0015672	monovalent inorganic cation transport	4.81E-04
	GO:0005506	iron ion binding	7.97E-04
			
**T5 - Rest of body/DOWN**			
	GO:0007156	homophilic cell adhesion	1.90E-05
	GO:0016337	cell-cell adhesion	2.83E-04
			
**T7 - Rest of body/DOWN**			
	GO:0070003	threonine-type peptidase activity	4.38E-12
	GO:0051603	proteolysis involved in cellular protein catabolic process	3.93E-14
	GO:0051444	negative regulation of ubiquitin-protein ligase activity	3.99E-05
	GO:0051443	positive regulation of ubiquitin-protein ligase activity	1.12E-04
	GO:0051439	regulation of ubiquitin-protein ligase activity involved in mitotic cell cycle	1.12E-04
	GO:0051438	regulation of ubiquitin-protein ligase activity	1.12E-04
	GO:0051437	positive regulation of ubiquitin-protein ligase activity involved in mitotic cell cycle	1.12E-04
	GO:0051436	negative regulation of ubiquitin-protein ligase activity involved in mitotic cell cycle	3.99E-05
	GO:0051352	negative regulation of ligase activity	3.99E-05
	GO:0051351	positive regulation of ligase activity	3.00E-04
	GO:0051340	regulation of ligase activity	3.00E-04
	GO:0044265	cellular macromolecule catabolic process	2.67E-11
	GO:0044257	cellular protein catabolic process	3.93E-14
	GO:0043632	modification-dependent macromolecule catabolic process	5.52E-10
	GO:0043234	protein complex	2.38E-08
	GO:0043161	proteasomal ubiquitin-dependent protein catabolic process	1.12E-04
	GO:0031398	positive regulation of protein ubiquitination	1.86E-04
	GO:0031397	negative regulation of protein ubiquitination	3.99E-05
	GO:0031396	regulation of protein ubiquitination	4.83E-04
	GO:0031145	anaphase-promoting complex-dependent proteasomal ubiquitin-dependent protein catabolic process	3.99E-05
	GO:0030163	protein catabolic process	8.38E-17
	GO:0022624	proteasome accessory complex	4.28E-08
	GO:0019941	modification-dependent protein catabolic process	5.52E-10
	GO:0019773	proteasome core complex, alpha-subunit complex	8.26E-04
	GO:0010498	proteasomal protein catabolic process	1.12E-04
	GO:0009057	macromolecule catabolic process	3.09E-09
	GO:0006511	ubiquitin-dependent protein catabolic process	4.88E-10
	GO:0006508	proteolysis	1.86E-04
	GO:0005839	proteasome core complex	4.38E-12
	GO:0005838	proteasome regulatory particle	6.90E-09
	GO:0004298	threonine-type endopeptidase activity	4.38E-12
	GO:0000502	proteasome complex	2.60E-29
			
**T7 - Rest of body/UP**			
	GO:0016491	oxidoreductase activity	9.14E-24
	GO:0055114	oxidation reduction	7.45E-20
	GO:0042302	structural constituent of cuticle	6.83E-13
	GO:0050662	coenzyme binding	1.18E-12
	GO:0048037	cofactor binding	1.18E-12
	GO:0050660	FAD or FADH_2 _binding	1.16E-08
	GO:0003824	catalytic activity	3.37E-08
	GO:0006066	alcohol metabolic process	2.52E-07
	GO:0016614	oxidoreductase activity, acting on CH-OH group of donors	2.52E-07

Statistically filtered transcriptional responses per *H. armigera *tissue (Welch's t-test, B&H FDR corrected P < 0.001) relative to gossypol-free diet expression data was subjected to GO enrichment analysis (P < 0.01) using Agilent GeneSpring GX11.5.1 software (Gossypol concentration: T5 = 0.016%, T7 = 0.16%).

At the higher gossypol concentration T7, the oxidoreductase molecular function was significantly enriched in both gut and RB. Among these genes several P450 enzymes are significantly upregulated, notably CYP6AE14 and CYP6AE11, while a few are downregulated (Figure 2). Several UGTs and carboxylesterases, and a fewer number of GSTs are upregulated, predominantly in the midgut (Figure 2). Many genes were down-regulated in G-T7; however, no particular GO enrichment was observed even if a less stringent p-value cut off (0.01) was applied to the analysis. Many ESTs down-regulated in the RB in the gossypol T7 treatment were found to be associated with the proteasome complex. (See additional file 5: Table S3 containing a list of differentially expressed genes across conditions with no GO enrichment.)

**Figure 3:**
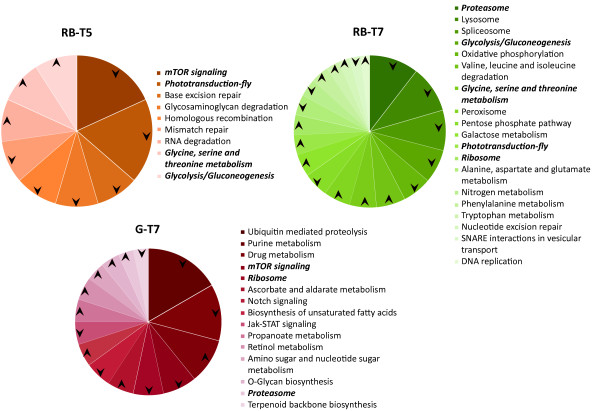
**KEGG pathway analysis on differentially regulated genes across gossypol dose-tissue experimental conditions**. Charts depict the proportion of genes, using fruit fly gene homology to *H. armigera *ESTs represented in the microarray, belonging to pathways occurring more or less frequently than expected in the test group relative to control (gossypol-free diet). Arrows indicate directionality of regulation for the corresponding genes in the pathway associated with significant z-scores (minimum number in gene set = 10) (Tissue: G = gut, RB = rest of body; Gossypol concentration: T5 = 0.016%, T7 = 0.16%).

Genes were also grouped with respect to biochemical pathways as categorized by KEGG for *D. melanogaster*, and those pathways statistically significant based on t-test comparisons along with the direction of the regulation based on the significant z-score obtained for the pathway are displayed in Figure 3 (See additional file 6: Table S4 listing the number of genes differentially expressed per KEGG category and the corresponding z-scores). The trends observed based only on this pathway analysis should be considered with some caution due to the low similarity scores of many of the *Drosophila *genes to their putative *H. armigera *orthologs. No KEGG pathways occurred more or less frequently than expected in the G-T5 condition when compared to the control. However, in the rest of the body, T5 affected the glycolysis/gluconeogenesis, amino acid metabolism and phototransduction pathways. The frequency of these same pathways was also distinct in RB-T7 and only the glycine, serine and threonine metabolic pathway was regulated in a similar way at both gossypol concentrations. The mammalian target of rapamycin (mTOR) pathway was similarly affected by T5 in the rest of the body and by T7 in the gut, with the down-regulation of genes observed more frequently than expected for this pathway. Only in G-T7, down-regulated genes are more frequent in the Notch and the JAK-STAT (Janus kinase-signal transducer and activator of transcription) signalling pathways, while genes involved in drug metabolism are up-regulated significantly. In turn, only in RB-T7, oxidative phosphorylation pathway genes are down-regulated while the peroxisome pathway is up-regulated (see additional file 7, Table S5, showing homologs in the peroxisome pathway). The proteasome and the ribosome pathways are affected in both tissues at gossypol T7 concentration but differentially regulated. Some other energy metabolism and gene regulation-related pathways were found uniquely for each condition when compared to the control, e.i. amino acid and sugar metabolic pathways in RB-T7. Consistencies between the GO and the KEGG pathway analyses are observed regardless of the low stringency for homology assignment in the case of the last analysis (e. g. proteasome, oxidation-reduction). We considered that the KEGG pathway analysis presented here, however, represents a first attempt to see transcriptional data under a regulatory framework that may allow us to direct our attention to testing additional hypotheses about gossypol effects in insects.

#### Rank Products Analysis

**Figure 4:**
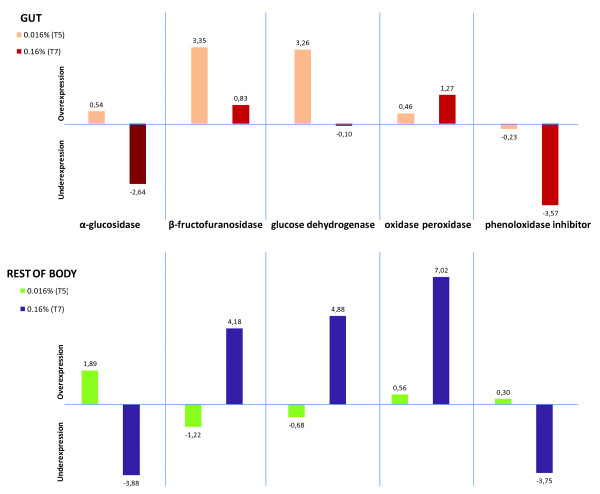
**Transcriptional response of energy acquisition and oxidative stress-related genes across experimental conditions**. Gene expression was found to be differentially regulated by the Rank Products method. The average of the normalized log-ratios across biological replicates for two probes representing each gene is plotted. The ratio is represented by either T5 (0.016%) or T7 (0.16%) over the control (0% gossypol) across tissues.

**Figure 5:**
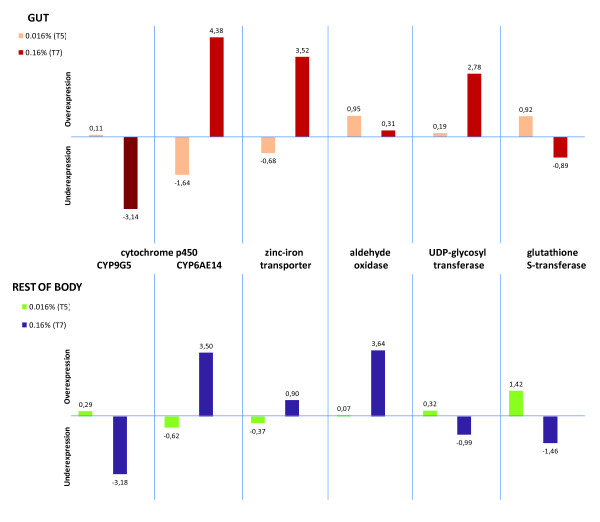
**Transcriptional response of a selection of putative detoxification genes across experimental conditions**. Gene expression was found to be differentially regulated by the Rank Products method. The average of the normalized log-ratios across biological replicates for two probes representing each gene is plotted. The ratio is represented by either T5 (0.016%) or T7 (0.16%) over the control (0% gossypol) across tissues.

The average of the normalized log-ratios across biological replicates was plotted for a set of selected genes ranked among the most up or down-regulated (See additional file 8: Table S6 containing the expression data of selected genes). Genes involved in energy acquisition such as β-fructofuranosidases and glucose dehydrogenases are among the most significantly up-regulated genes in G-T5 (Figure 4). Among the most up-regulated genes are also sugar-degrading enzymes in the RB (i.e. α-glucosidases). Contrary to their up-regulation in T5, α-glucosidase genes are among the most significantly down-regulated in both tissue types at T7 along with other glycoside hydrolases in the RB at this same gossypol dose. However, β-fructofuranosidase is up-regulated in the RB-T7 experimental condition and homologs of phenoloxidase inhibitor and oxidase peroxidase genes are differentially regulated in both tissues at this same concentration. P450 s are ranked among the most up and down-regulated genes in both tissues for each gossypol concentration (Figure 5). At T5 in the gut, a slight up-regulation of glutathione transferase GST24 is observed, while zinc-iron transporters are ranked among the most up-regulated genes in the G-T7 condition. (See additional files 9, 10, 11, 12, 13, 14, 15, 16 for complete lists of microarray probes ranked for up and down-regulation across experimental conditions.)

#### Quantitative real-time PCR

Additionally, we inspected the gene expression of CYP6E14 and CYP6AE11 (with 91% nucleotide identity to CYP6E14) across all gossypol concentrations by qRT-PCR (See additional file 17 Figure S3) CYP6AE14 and CYP6AE11 are highly up-regulated at the highest gossypol concentration. CYP6AE11 expression declines slightly over 0.004 to 0.016% gossypol concentrations compared to the control 0%, and then increases at the highest concentration, roughly mirroring the hormetic growth response.

## Discussion

### Effect of gossypol on larval growth

The biphasic response to gossypol concentration is similar to observations for another heliothine species, *Heliothis virescens *[13] where the highest larval weight was obtained when the insects were exposed to a 0.0125% gossypol-containing diet. Before our study, the effect of gossypol doses lower than 0.0125% on larval development had not been examined. The maximum stimulatory concentrations of gossypol on weight gain in heliothine larvae may lie between 0.008% and 0.04%. Data in Figure 1 are also consistent with the results of Stipanovic et al. [25] where the developmental effect of 0.16% gossypol and higher towards *Helicoverpa zea *larvae resulted in a significant extension of the time to reach pupation and decrease of pupal weight regardless of gossypol enantiomer or racemate used in the diet. The results herein indicate that the inhibitory effects of gossypol on *H. armigera *can be observed even if the insect is exposed later in its development, since we did not rear the insects on the gossypol-containing diet from the neonate stage as done by other researchers [13,25,32]. In addition, a hormetic effect on larval weight gain can also be observed on newly molted fifth instar larvae even after three days of exposure to gossypol, although in this regard we have no data on the relative effect of the racemic mixture versus the separate enantiomers. Thus hormesis seems to be a general feature of the response of generalist heliothines to gossypol. The many host-plant specialists within this insect group, which do not normally encounter gossypol in their diet, have not been tested for hormetic effects.

### Coping with gossypol

It has been suggested that bacterial infection stimulates epithelial renewal via reactive oxygen species (ROS) production [33]. A similar stimulatory response may be triggered by low gossypol concentrations in the CBW gut, since a consequence of gossypol toxicity may involve the generation of superoxide free radicals damaging the epithelium [5]. However, if present, such a proliferative response does not seem to be mediated by significant transcriptional changes in the midgut at the low gossypol concentration.

Gossypol, as a lipophilic compound, interacts with cellular membranes and forms bonds with the amine groups of proteins via Schiff's base formation; cross-linking of membrane proteins has been suggested to block cell-to-cell communication [34]. Previously, it has been proposed that adaptive changes in lipid content can occur upon gossypol exposure [34,35]. We speculate that this effect on genes involved in cell adhesion may represent a response to the cytotoxicity of gossypol at this concentration. A down-regulation of cell adhesion genes has also recently been observed as a consequence of gossypol exposure in mammalian cells [36]. A 12-cadherin domain protein is expressed in the midgut of lepidopteran larvae where it is a binding target for Cry1A toxins from *Bacillus thuringiensis *(Bt) [37]. It has recently been shown that the same cadherin is also expressed in the larval testis of the cotton specialist pink bollworm (*Pectinophora gossypiella*) suggesting a role in sperm production [38]. Mutations in this protein responsible for Bt toxin resistance reduce fertility somewhat and in turn Bt-resistant pink bollworm is more susceptible to gossypol [39]. We observed down-regulation of the homologous 12-domain cadherin protein, not in the midgut where it abundantly expressed but in the rest of the body which includes the testis. In vertebrates, some cadherins are known to interact with compounds leading to signal transduction events such as the carcinoembryonic antigen-related cell adhesion molecules (CEACAMs); however insect homologs of the latter are unknown.

The mode of action of gossypol has been studied mostly in relation to its toxic or therapeutic effects on mammalian cells. These studies show that certain low doses of gossypol can have antitumor, antiviral and antiparasitic activities mostly due to the fact that gossypol inhibits key proteins belonging to different classes of enzymes such as oxidoreductases, hydrolases and transferases [5]. Gossypol is not considered a bactericidal compound [40]. Therefore, we do not consider that the hormetic effect observed at T5 is a consequence of gossypol acting as a toxin to bacteria possibly present in the artificial diet.

A stimulatory effect on energy metabolism due to gossypol has been observed in cultured mouse cells. Relatively low doses of gossypol were added to the cell medium along with glucose resulting in an increase of lactate production. Additionally, the inhibition of oxygen consumption produced by 5 mM glucose was reversed when gossypol was added to the medium [41]. The phenomenon was explained by the ability of gossypol to uncouple oxidative phosphorylation, leading to a decrease of mitochondrial production of adenosine triphosphate (ATP). Thus, glycolysis and the production of lactate through the Embden-Meyerhof pathway were stimulated as an attempt to maintain the required ATP levels in the cell. The authors concluded that the response towards gossypol may depend on the cell's glycolytic and mitochondrial oxidative phosphorylation capacity and on its ability to maintain acid-base homeostasis [41]. A differential susceptibility and hormetic effect in oxygen production has also been observed between somatic and germ rat cells exposed to different concentrations of gossypol [42]. Therefore, it is possible that the gut epithelium can be maintained at low gossypol doses and even stimulated by the uncoupling of mitochondria. This may explain the fact that we do not see the biological process of glycolysis enriched by differentially regulated genes since the response to gossypol may depend on subtle adjustments in metabolism which take place as acclimatization at the protein level. However, glycolysis-related genes were affected in the RB at both gossypol concentrations examined.

It has previously been described that β-fructofuranosidases of *Bombyx mori *are resistant to inhibition by mulberry sugar mimic alkaloids which inhibit α-glucosidases, and therefore represent an adaptation to the mulberry host in this specialist moth [43]. If α-glucosidases are also inhibited by gossypol, the up-regulation of β-fructofuranosidase genes by the high dose of gossypol in *H. armigera *might be explained by a similar compensatory mechanism. Interestingly, we found that in *H. armigera*, the regulation of at least one β-fructofuranosidase gene (GH32FruA-1), in response to gossypol, is tissue and dose dependent. The same seems to be true for glucose dehydrogenase genes, which are up-regulated at gossypol T7 only in the RB.

Cross-linking amino acids and proteins, phenolic compounds cause oxidative damage to the midgut cells of insects [44]. Thus, the up-regulation of peroxisome-related genes may be part of an antioxidant response to the ROS generated by gossypol activity at such high concentration. Consistently, phenoloxidase inhibitor and oxidase peroxidase genes are respectively down-and up- regulated in both tissues, indicating that homeostasis has been compromised possibly due to major tissue wounding caused by oxidative stress.

The proteasome apparatus, composed of a proteolytic core and two regulatory particles, degrades a variety of cellular proteins involved in many essential functions, such as signal transduction pathways, stress signaling, inflammatory responses, and apoptosis. If increased proteolysis of cellular targets inactivated by binding to gossypol were required, we would expect an up-regulation of this system. The fact that the proteasome is instead down-regulated by gossypol suggests that the response of CBW to this compound may be similar to the response by an annelid towards fluoranthene, a polycyclic aromatic hydrocarbon, known to disrupt biological membranes due to its lipophilicity [45], characteristics shared with the gossypol molecule. This down-regulation of the ubiquitin-26S proteasome pathway is interpreted as a mechanism to reduce the proteolytic turnover of the aryl hydrocarbon receptor (Ahr) which is considered a mediator in the expression of genes involved in detoxification. In mammals, Ahr regulates cellular responses to certain polycyclic aromatic hydrocarbon toxins similar to fluoranthene and gossypol [45]. Several pathways also mediating gene expression (i.e. mTOR, JAK-STAT, phototransduction and Notch) were affected, representing potential signaling pathways involved in the response towards gossypol which deserve further attention.

### Gossypol detoxification

Since gossypol is a defensive chemical encountered in some but not all of the hostplants of this generalist herbivore, some transcriptional responses are expected to be directed towards its detoxification, and these can be compared with previous metabolic studies. The metabolic fate of gossypol in *Heliothis virescens *larvae has been examined by means of ^14^C-labelling the compound and adding it to an artificial diet. 25% was found to be metabolized by conjugation with six sugar molecules per mole of gossypol and excreted in the frass, whereas glutathione conjugates were not detected [46]. This is consistent with the up-regulation of 10 UDP-glycosyltransferases but only one glutathione transferase in the midgut. If endogenous α-glucosidases were capable of hydrolyzing these glucose conjugates, their down-regulation would be favored in favor of up-regulation of β-fructofuranosidases which can still digest carbohydrates. About 33% of the labelled gossypol was found in the larval tissues, mostly in the fat body and the rest of the compound excreted as free gossypol or bound to components of the frass. About 10% of labelled gossypol was recovered as carbon dioxide but the mechanism has not been elucidated in this species. A similar study done with rats revealed that decarbonylation of gossypol is an important detoxification pathway; labeled ^14^CO_2 _appeared in expired air 1 hour after feeding of ^14^C-gossypol [47]. However, in swine decarbonylation may not be the main detoxification pathway since the main products found were glucuronides, sulphates and unconjugated metabolites [48]. These contrasts between species made the authors consider whether the degree of decarbonylation of gossypol indicates the susceptibility towards the allelochemical (i.e. rats, more tolerant to this phenolic compound, retain less of it in their tissues and detoxify it mostly by decarbonylation) [48].

A zinc-iron transporter was upregulated in the gut at T7 (Figure 5), which is consistent with gossypol acting as a sink for iron in the midgut lumen. Ferric ions have been shown to precipitate gossypol, while ferrous ions produce a soluble chelate which is precipitated by calcium [3]. There is evidence that at least some of the gossypol decarbonylation observed in the rat digestive tract occurs by an auto-oxidation process catalyzed by ferrous ions through a free-radical chain mechanism similar to that proposed for benzaldehyde decarbonylation [47].

The oxidoreductase molecular function in the G and RB is one of the most significantly enriched at T7 and within this category P450 enzymes are prominent. In order to metabolize gossypol to gossic acid several oxidation steps are required [48], for which the P450 s are candidates. Consistent with previous results by others [21], CYP6AE14 is up-regulated in the G and the RB at T7. However, CYP6AE14 is slightly down-regulated at T5 in both tissues. Moreover, 13 additional P450 s are upregulated and two downregulated in the gut at the highest gossypol concentration. When CYP6AE14 was silenced in *H. armigera *by feeding dsRNA or transgenic cotton transformed with a construct expressing dsRNA, larval growth was greatly reduced in the presence of gossypol [21,22], however there are no heterologous expression studies providing information about CYP6AE14 substrate specificity and its direct role in gossypol metabolism. Racemic gossypol promotes the formation of superoxide free-radicals when incubated with rat liver microsomes [49]. There is evidence indicating that the damage caused by these superoxide free radicals is due to the interaction of gossypol with the iron of the P450 enzyme [5]. It might be worth testing whether CYP6AE14 is more susceptible to this effect which may provide an alternative explanation for its up-regulation.

The strong upregulation of several carboxylesterases in the midgut poses somewhat of an enigma, since neither gossypol nor any of its known metabolites are suitable substrates for esterases. In some cases, overexpression of esterases confers resistance to organophosphorus insecticides, due to sequestration rather than metabolism (reviewed in [50]). If some of these esterases similarly trap gossypol, keeping it from more sensitive cellular targets, this could be an effective but expensive mechanism of tolerance. Alternatively, gossypol may interact with a regulatory molecule such as Ahr which controls a suite of different detoxicative genes, only some of which are directly involved in the detoxification of a given xenobiotic.

## Conclusion

### Responses to low concentrations

Low concentrations of gossypol could stimulate larval growth by increasing food consumption rate, increasing food conversion efficiency, or reducing the metabolic cost of other physiological activities. Cotton specialists that can efficiently deal with gossypol could be selected to use it as a feeding stimulus, as glucosinolates are by crucifer specialists such as *Plutella xylostella *[51]. If these responses were caused solely by gossypol's interacting with proteins present in the cell without causing any changes in gene expression, they would go undetected by our approach. Moreover, such responses are unlikely to have been shaped by natural selection on the herbivore specifically to benefit from low concentrations of gossypol; because such low concentrations are rarely encountered in nature.

In evaluating whether transcriptional responses can shed any light on the mechanism of hormesis of gossypol, it is worth remembering that this compound is made by the cotton plant and stored in special glands primarily to deter herbivory, and that *H. armigera *frequently uses cotton as a host but also consumes many other plants that do not make gossypol. The first plant consumed by a larva is dictated by its mother's oviposition choice, and there is usually limited scope for larval movement to another plant after that. We would therefore expect that natural selection would favor inducible, adaptive responses by the insect to gossypol when it is first consumed, and that many of these responses would be harmful, or at least not beneficial, in the absence of gossypol; otherwise they would be constitutively expressed. This history of coevolutionary interaction is an important difference to most studies of gossypol's effects on mammalian systems; or indeed most studies exhibiting a hormetic effect of any kind, which usually deal with pollutants or drugs that do not have an evolutionary history with the target system.

Beneficial effects of a toxin at low doses are usually attributed to completely different mechanisms than those by which the toxin exerts its ill effects at high doses. These beneficial effects are either completely unpredictable, or fall into a general pattern of activating stress-response mechanisms that are generally beneficial to the organism. However, if they were always beneficial, and incurred no additional costs, natural selection would favor their constitutive expression. Therefore one of the paradoxes of hormesis that is often overlooked is why the organism should require slight stresses to increase its fitness. Rather than having a general explanation, whether or not hormesis is observed would seem to be highly system-dependent and the precise nature of such benefits unpredictable.

### The evolution of hormesis

The history of cotton-feeding insects' interaction with cotton, however, does potentially offer an evolutionary explanation for hormetic effects of a specific plant defensive compound on a generalist herbivore. We propose that different types of transcriptional responses to dietary gossypol should have different thresholds, which are shaped by natural selection. Detoxicative mechanisms that are specific to gossypol are likely to be ernegetically costly, and even cause cellular damage such as the generation of reactive oxygen species by unproductive P450 reactions. Additional stress-coping mechanisms that respond to damage directly caused by gossypol and by its detoxification are also expected to be costly. Since gossypol is either completely absent from the hostplant or present in high concentrations; such responses to high gossypol concentrations that are specific to that compound should be selected for. Conversely, highly sensitive induction of detoxicative responses at low gossypol concentrations would not be selected for, and in fact selected against if they trigger inappropriate and costly responses. However, mildly beneficial responses to gossypol that impose modest energetic costs would not be subject to similar selection for a higher threshold. They should even be selected to be induced at effectively low concentrations if initially high gossypol concentrations are reduced by metabolism.

The extremely wide diversity of systems in which hormesis has been observed makes it unlikely that a single general hypothesis could satisfactorily account for them all. However, in *H. armigera *hormesis emerges as one of two types of specific transcriptional adaptations to gossypol, not merely as a general response to low levels of stress. Gossypol itself exerts no ill effects at unrealistically low concentrations which are still sufficient to induce low-cost growth-promoting adaptations to gossypol. Only at higher concentrations does the damage caused by gossypol and mechanisms that cope with it overcome these benefits. We would expect the same reasoning to apply to any generalist herbivore that has developed adaptations to several different plant toxins, only one of which may be encountered in an individual's lifetime. Hormesis in such systems can thus be seen as one end of a spectrum of plant-insect coevolutionary interactions.

## Authors' contributions

HV constructed *H. armigera *cDNA libraries, sequenced ESTs and performed the EST sequence analysis. MPCM and SJA participated in the conception and design of the study. MPCM recorded most of the developmental data and processed the *H. armigera *tissue samples for RNA isolation, labeling and hybridization onto microarrays and was responsible for analysis of microarray data. SJA was responsible for the statistical analysis of the developmental data. DGH reviewed the microarray analysis, generated and curated contig assemblies. MPCM drafted the manuscript and together with SJA, HV and DGH contributed to the interpretation and the iterative refinement of the article. All authors have read and approved the submitted version.

## Supplementary Material

Additional file 1 **Table S1**. Best RefSeq homolog hits from *Drosphila melanogaster, Tribolium castaneum *and *Bombyx mori *to *H. armigera *sequences. Click here for file

Additional file 2 **Figure S1**. Effect of gossypol on larval developmental time to pupation. The Log (mg/L) gossypol was plotted against larval developmental time to pupation (days). Means that are not connected by the same letter are significantly different from each other as determined by post-hoc Duncan test (P < 0.05) (feeding treatments T5 = 0.016% and T7 = 0.16% gossypol in insect diet). Click here for file

Additional file 3 **Figure S2**. Microarray design and hierarchical clustering of experimental conditions. A: Two-color double reference design followed for microarray hybridizations for each tissue. B: Hierarchical clustering determining the relationship between the samples belonging to the gossypol concentration experimental conditions per each tissue. Gut = G; rest of body = RB. Click here for file

Additional file 4 **Table S2**. Cell-adhesion gene probes down-regulated upon 0.016% gossypol in the *H. armigera *rest of larval body. The Genbank accession number corresponds to the best Blast2go hit by the corresponding *H. armigera *EST represented by the probe in the microarray. Click here for file

Additional file 5 **Table S3**. Genes differentially expressed across experimental conditions without Gene Ontology enrichment. List of genes found to be differentially expressed in *H. armigera *gut (G) and rest of body (RB) larval tissue to different gossypol concentrations in diet (T5 = 0.016%; T7 = 0.16%) relative to the control (CT = 0%) but with no Gene Ontology enrichment. Differential expression in response to gossypol identified using a Welch's t-test, B&H FDR P < 0.001. Click here for file

Additional file 6 **Table S4**. KEGG pathway analysis on differentially regulated genes across gossypol dose-tissue experimental conditions. Based on homology, we inspected *Drosophila melanogaster *KEGG pathway enrichment in the *H. armigera *transcriptional data for each t-test comparison of gossypol dose (T5 or T7) relative to control (CT) per tissue. z-score statistics were applied in GeneSifter^® ^to determine whether a pathway occurs more or less frequently than expected. Click here for file

Additional file 7 **Table S5**. Peroxisome KEGG pathway gene probes up-regulated by 0.16% gossypol in the *H. armigera *larval body. KEGG pathway analysis was based on gene homology established by obtaining best BLAST hits for *H. armigera *ESTs to *D. melanogaster *genes. Differential expression is relative to control (gossypol-free diet). Click here for file

Additional file 8 **Table S6**. Transcriptional responses of selected genes across experimental conditions. Normalized log-ratios across biological replicates for each treatment are compared to the control for a selection of genes found to be differentially expressed by the Rank Products method. Accession number, gene description and best hit homolog to *D. melanogaster *Refseq nucleotide and protein is included. The average of two probes per gene was used for its graphical display in Figures 4 and 5. Click here for file

Additional file 9 **Table S7_T5_G_RPlist_up**. Rank Products list detecting differentially up-regulated genes in the G-T5 treatment. The most significantly up-regulated genes are at the top of the list. Click here for file

Additional file 10 **Table S8_T5_G_RPlist_down**. Rank Products list detecting differentially down-regulated genes in the G-T5 treatment. The most significantly down-regulated genes are at the top of the list. Click here for file

Additional file 11 **Table S9_T5_RB_RPlist_up**. Rank Products list detecting differentially up-regulated genes in the RB-T5 treatment. The most significantly up-regulated genes are at the top of the list. Click here for file

Additional file 12 **Table S10_T5_RB_RPlist_down**. Rank Products list detecting differentially down-regulated genes in the RB-T5 treatment. The most significantly down-regulated genes are at the top of the list. Click here for file

Additional file 13 **Table S11_T7_G_RPlist_up**. Rank Products list detecting differentially up-regulated genes in the G-T7 treatment. The most significantly up-regulated genes are at the top of the list. Click here for file

Additional file 14 **Table S12_T7_G_RPlist_down**. Rank Products list detecting differentially down-regulated genes in the G-T7 treatment. The most significantly down-regulated genes are at the top of the list. Click here for file

Additional file 15 **Table S13_T7_RB_RPlist_up**. Rank Products list detecting differentially up-regulated genes in the RB-T7 treatment. The most significantly up-regulated genes are at the top of the list. Click here for file

Additional file 16 **Table S14_T7_RB_RPlist_down**. Rank Products list detecting differentially down-regulated genes in the RB-T7 treatment. The most significantly down-regulated genes are at the top of the list. Click here for file

Additional file 17 **Figure S3**. CYP46AE14 and CYP46AE11 expression levels across gossypol treatments as measured by qRT-PCR. Click here for file
