# Supplementary figures and images for: Transcriptional responses underlying the hormetic and detrimental effects of the plant secondary metabolite gossypol on the generalist herbivore Helicoverpa armigera
Source: BMC Genomics. 2011 Nov 23;12:575. doi: 10.1186/1471-2164-12-575 (PMC3235194; doi:10.1186/1471-2164-12-575)

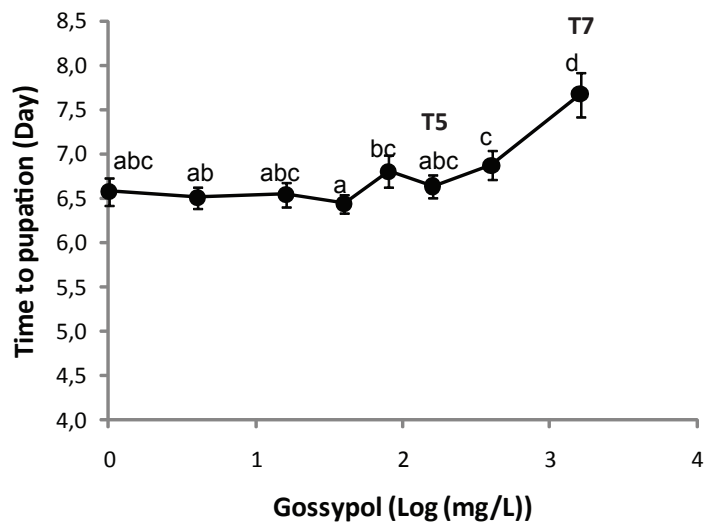

Supplement: Additional file 2 — Figure S1. Effect of gossypol on larval developmental time to pupation. The Log (mg/L) gossypol was plotted against larval developmental time to pupation (days). Means that are not connected by the same letter are significantly different from each other as determined by post-hoc Duncan test (P < 0.05) (feeding treatments T5 = 0.016% and T7 = 0.16% gossypol in insect diet). [file 1471-2164-12-575-S2.PDF]

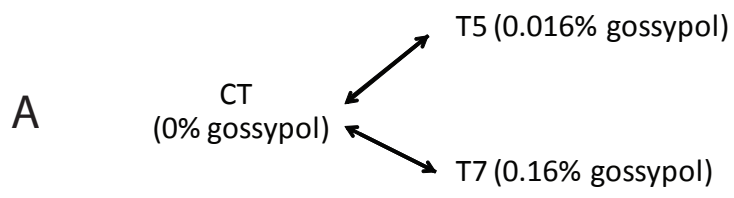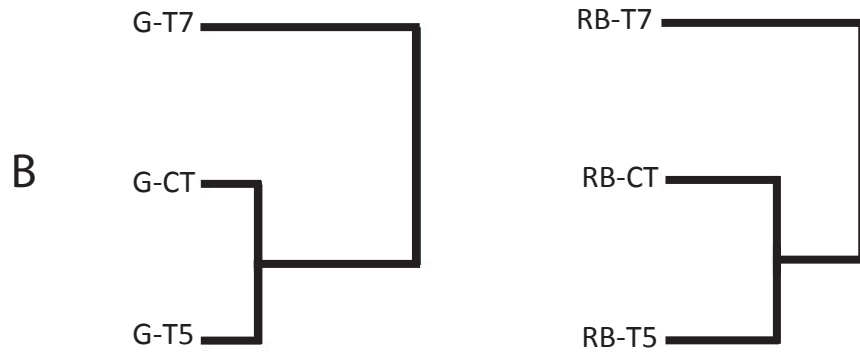

Supplement: Additional file 3 — Figure S2. Microarray design and hierarchical clustering of experimental conditions. A: Two-color double reference design followed for microarray hybridizations for each tissue. B: Hierarchical clustering determining the relationship between the samples belonging to the gossypol concentration experimental conditions per each tissue. Gut = G; rest of body = RB. [file 1471-2164-12-575-S3.PDF]

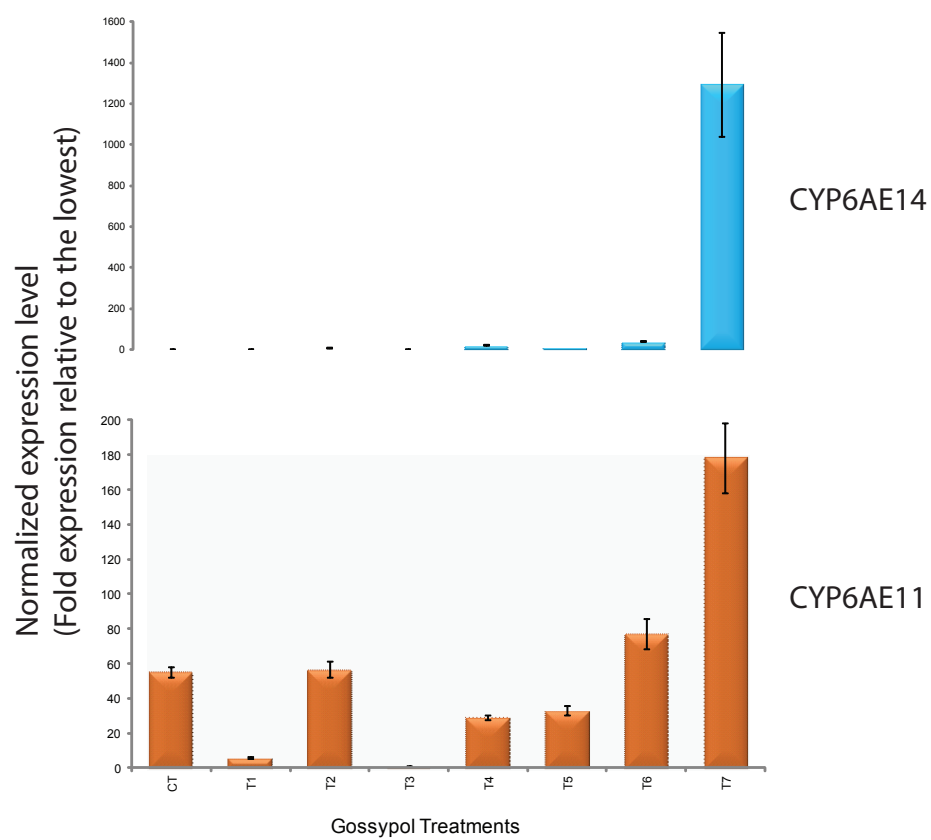

Supplement: Additional file 17 — Figure S3. CYP46AE14 and CYP46AE11 expression levels across gossypol treatments as measured by qRT-PCR. [file 1471-2164-12-575-S17.PDF]
